# Non-Hermitian metasurface with non-trivial topology

**DOI:** 10.1515/nanoph-2021-0731

**Published:** 2022-02-16

**Authors:** Frank Yang, Ciril S. Prasad, Weijian Li, Rosemary Lach, Henry O. Everitt, Gururaj V. Naik

**Affiliations:** Department of Electrical & Computer Engineering, Rice University, Houston 77005, TX, USA; Applied Physics Graduate Program, Smalley-Curl Institute, Rice University, Houston 77005, TX, USA; U.S. Army DEVCOM Army Research Laboratory-South, Houston, TX, USA

**Keywords:** exceptional concentric ring, metasurface, non-Hermitian, plasmonics, topological photonics

## Abstract

The synergy between topology and non-Hermiticity in photonics holds immense potential for next-generation optical devices that are robust against defects. However, most demonstrations of non-Hermitian and topological photonics have been limited to super-wavelength scales due to increased radiative losses at the deep-subwavelength scale. By carefully designing radiative losses at the nanoscale, we demonstrate a non-Hermitian plasmonic–dielectric metasurface in the visible with non-trivial topology. The metasurface is based on a fourth order passive parity-time symmetric system. The designed device exhibits an exceptional concentric ring in its momentum space and is described by a Hamiltonian with a non-Hermitian $Z_{3}$ topological invariant of *V* = −1. Fabricated devices are characterized using Fourier-space imaging for single-shot *k*-space measurements. Our results demonstrate a way to combine topology and non-Hermitian nanophotonics for designing robust devices with novel functionalities.

## 1 Introduction

In recent years, intense research efforts have been undertaken to understand the roles of symmetry and topology in non-Hermitian physics, with photonics acting as a convenient testbed [1, 2]. Manipulating topology in photonics is enticing due to the promise of devices that are robust against defects. Such devices would have behaviors encoded in their topology rather than their geometry. Already, topological photonics in Hermitian systems has yielded remarkable demonstrations of unidirectional edge states [3–5] and topological photonic insulators [6–8]. Combining topology and non-Hermitian physics holds further promise for the observation of rich physical phenomena [9–12].

Non-Hermiticity in photonics entails the conscious introduction of gain and loss in optical systems, extending design of permittivity/permeability to the complex plane. Non-Hermitian Hamiltonians are not guaranteed to have real eigenvalues, orthogonal eigenmodes, or physically meaningful observables – unlocking a host of physical effects without Hermitian counterparts. One such unique feature is the exceptional point. Exceptional points are non-Hermitian energy degeneracies at which eigenmodes skew together and render the system defective [13–15]. Behaviors near exceptional points have resulted in demonstrations of interesting phase transitions [16, 17], unidirectional invisibility [18–20], ultrasensitive sensing [21, 22], single mode lasing [23, 24], and chirality [25–27]. The topology of self-intersecting Riemann sheets associated with exceptional points has also enabled demonstrations of chiral mode conversion [28, 29].

The interplay between topological and non-Hermitian photonics has resulted in observation of topological edge states in non-Hermitian lattices [30–32], topological insulator lasers [33, 34], and light funneling by the non-Hermitian skin effect [35]. Topologically non-trivial features in momentum space, including exceptional rings [36–38] and bulk Fermi arcs [39, 40], have also been reported.

To date, demonstrations of topological and non-Hermitian photonics have been largely confined to super-wavelength scales. This is because of increased radiative losses at deep-subwavelength scales. Additionally, schemes for observing higher-order (*N* > 2) parity-time symmetry on the nanoscale have remained elusive [41]. Here, we address both of these challenges. In this work, we carefully design radiative losses at the nanoscale to demonstrate a non-Hermitian plasmonic–dielectric metasurface at visible wavelengths with non-trivial topology. Our device exhibits higher order (*N* > 2) parity-time symmetry and makes use of 4 modes to unlock interesting topological features. The metasurface displays an exceptional concentric ring in its momentum space. The observed exceptional concentric ring indicates a non-Hermitian $Z_{3}$ topological invariant of *V* = −1 when the system is modeled with an equivalent Hamiltonian. The device is investigated with an analytical model, full-wave simulations, and experimentally. We use Fourier plane imaging and spectroscopy to characterize photonic bandstructure of fabricated devices efficiently. Our results hold promise for the design of nanophotonic devices with directional response and topological protection.

## 2 Design of topologically non-trivial, non-Hermitian metasurface

Our non-Hermitian metasurface design is based on the principles of passive parity-time (PT) symmetry [16, 42], [43], [44]. Passive PT-symmetric systems are a subset of non-Hermitian Hamiltonians that can exhibit fully real eigenvalues [45], exceptional points, and phase transitions without the introduction of gain [16]. A simple example of such a system contains a lossless resonator coupled to a lossy resonator [42, 44]. For low coupling between resonators, the real parts of the eigenvalues are degenerate while the imaginary parts are split (PT-broken regime). Increasing coupling can bring the coupled resonators to an exceptional point, where eigenvalues are fully degenerate and system dimensionality decreases. Further increasing coupling brings the system to the PT-symmetric regime, where the real parts of the eigenvalues split and the imaginary parts are degenerate.

**Figure 1:**
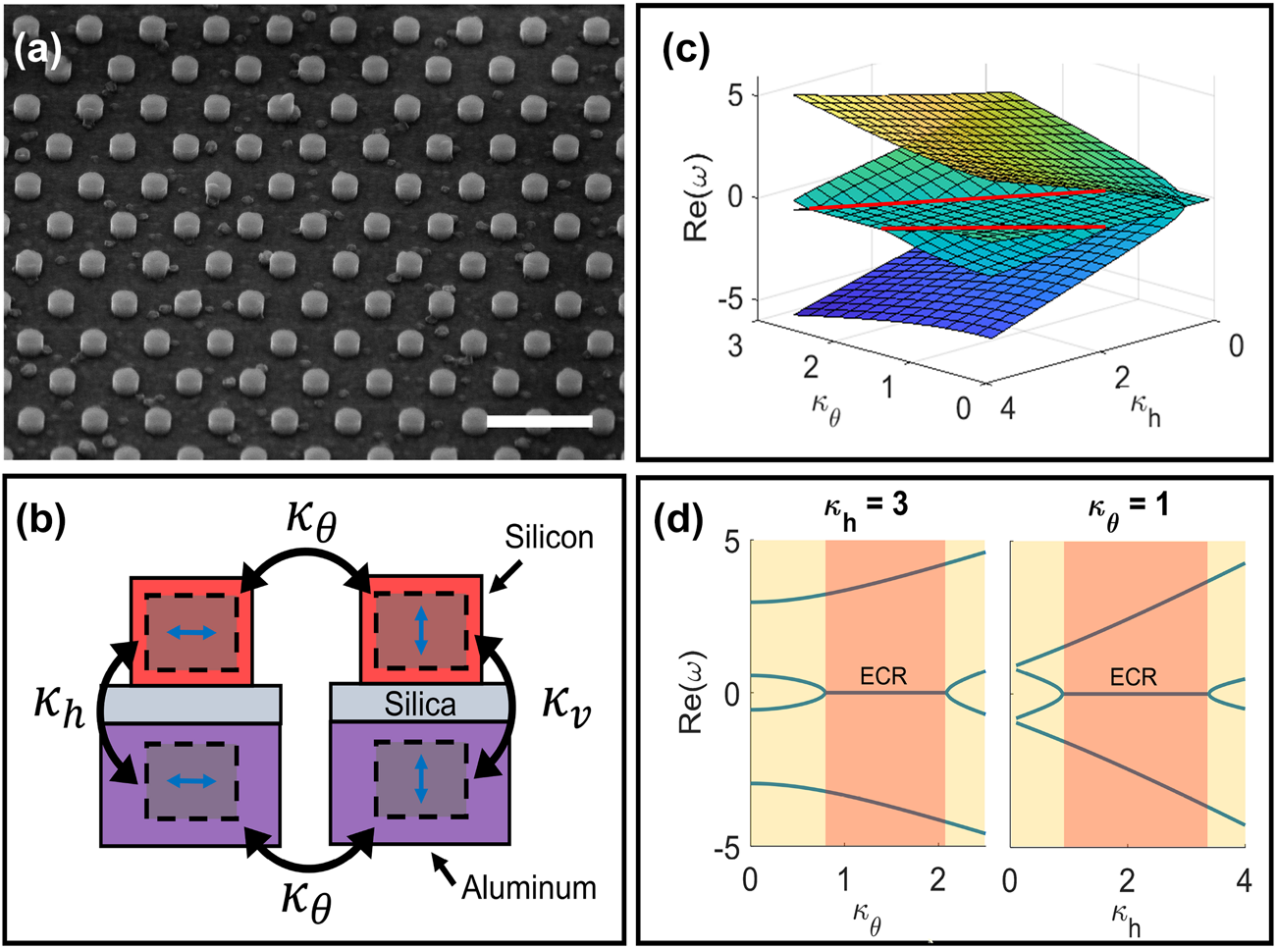
Ground plane system: coupling between horizontal and vertical modes unlocks topological effects. (a) Scanning electron microscope image of non-Hermitian metasurface (scale bar: 1 μm). Silicon nanocylinders (radius = 120 nm, height = 120 nm) are arranged in a hexagonal lattice (lattice constant = 600 nm) on a silica spacer layer (variable thickness) and aluminum layer. (b) The metasurface contains horizontal and vertical photonic modes coupled to their respective image charges in the aluminum ground plane (*κ*_h_, *κ*_v_). Horizontal and vertical modes can couple to each other by varying the angle of incidence of excitation (*κ*_*θ*_). (c) Real part of eigenvalues for representative 4 × 4 Hamiltonian of the coupled system, showing exceptional lines (marked in red). (d) Cross sections of real part of eigenvalues, holding either *κ*_h_ or *κ*_*θ*_ constant. The exceptional concentric ring (red, labeled) and PT-symmetric (yellow) phases are accessible by varying either parameter (*κ*_h_, *κ*_*θ*_) independently.

Typically, radiative losses decrease achievable quality factors for deep-subwavelength nanostructures. Here, we make use of design principles established in previous works to control radiative losses while varying absorption losses, thereby achieving a nanoscale passive PT-symmetric device [42–44]. The metasurface device is composed of silicon nanocylinders in a hexagonal array, which sits atop a silica spacer layer and aluminum ground plane (shown in Figure 1(a)). Silicon nanocylinders have height = 120 nm, radius = 120 nm, and lattice constant = 600 nm. The silicon nanocylinder array supports a horizontally oriented quasi-bound state in the continuum (BIC) mode [44, 46], which represents our lossless resonator. The quasi-BIC arises from a local resonance in the bandgap of the array of Si disks. The bandgap restricts radiative losses, resulting in highly localized modes in the sub-wavelength resonators.

This photonic mode couples to its image charges in the aluminum ground plane under normally incident input excitation (Figure 1(b), left). The image charges in aluminum represent the lossy resonator. Additionally, by varying the out-of-plane angle of incidence for a transverse magnetic (TM) input wave, exciting a vertically oriented dielectric mode becomes possible (Figure 1(b), right). The vertical photonic mode also couples to its corresponding plasmonic mode in the aluminum. This set of four resonators is described by the 4 × 4 Hamiltonian given in Eq. (1). Due to absorption in silicon in the near-IR, the dielectric modes considered are not strictly lossless. They are effectively modeled as lossless. Eigenvalue evolution in the parameter space ultimately depends on loss asymmetry between dielectric and plasmonic modes, not the dielectric loss.

(1) $$\hat{H}=\left[\begin{array}{cccc} \omega_{\text{h}}+i\gamma_{\text{h}1} & \kappa_{\text{h}} & \kappa_{\theta } & 0\\ \kappa_{\text{h}} & \omega_{\text{h}}+i\gamma_{\text{h}2} & 0 & \kappa_{\theta }\\ \kappa_{\theta } & 0 & \omega_{\text{v}}+i\gamma_{\text{v}1} & \kappa_{\text{v}}\\ 0 & \kappa_{\theta } & \kappa_{\text{v}} & \omega_{\text{v}}+i\gamma_{\text{v}2} \end{array}\right]$$

Here, *ω*_h_ and *ω*_v_ represent resonance frequencies of horizontal and vertical modes. Photonic and plasmonic modes are labeled by 1 (*γ*_h1_, *γ*_v1_) and 2 (*γ*_h2_, *γ*_v2_), respectively. *κ*_h_, *κ*_v_, and *κ*_*θ*_ represent horizontal plasmonic–photonic coupling, vertical plasmonic–photonic coupling, and horizontal–vertical coupling, respectively. *γ*_h1_, *γ*_h2_, *γ*_v1_, and *γ*_v2_ are resonator loss rates. Horizontal and vertical photonic modes are spatially and spectrally overlapped in single silicon resonators (coupled by *κ*_*θ*_) by design. Horizontal and vertical plasmonic modes are also spatially overlapped (coupled by *κ*_*θ*_). Coupling between horizontal and vertical modes (*κ*_*θ*_) can be non-zero and is controlled by the out-of-plane angle of excitation. This is because horizontal and vertical modes are not fully orthogonal. They are primarily electric dipole in nature, but are, in reality, hybrid electric and magnetic dipole modes. The silica spacer thickness controls the coupling between the lossless photonic modes and the lossy plasmonic modes (*κ*_h_, *κ*_v_). Hence, *κ*_h_ and *κ*_v_ do not vary independently.

Figure 1(c) plots the real part of the eigenvalue spectrum of the 4 × 4 Hamiltonian given in Eq. (1) while sweeping *κ*_h_, *κ*_v_, and *κ*_*θ*_. The imaginary part of eigenvalues is provided in Figure S1 of the Supplementary Material. Because *κ*_h_ and *κ*_v_ are both determined by the silica spacer thickness, we set *κ*_v_ = 0.25*κ*_h_. Additionally, we set *γ*_h1_ = *γ*_v1_ = 0 (lossless photonic modes) and *γ*_h2_ = *γ*_v2_ = 1 (lossy plasmonic modes). Three regions of interest appear in the eigenvalue spectrum. First, when *κ*_h_ and *κ*_*θ*_ are close to zero, the real components of the four eigenvalues are degenerate while the imaginary components are split. As *κ*_h_ or *κ*_*θ*_ are increased, the degeneracy is lifted for the real part of two eigenvalues, leaving a sheet of two-fold degeneracy. If *κ*_h_ or *κ*_*θ*_ are increased further, the degeneracy of the real part of eigenvalues is fully lifted, and all four imaginary parts are degenerate. The transition between the second and third regions occurs at an exceptional point, which forms two exceptional lines seen at large *κ*_h_ or *κ*_*θ*_ values (marked in red). Exceptional lines hint at the possibility of non-trivial topology in the metasurface.

## 3 Results and discussion

To analyze the topology of our exceptional point system, we use the mathematical framework developed by Wang et al. [37]. We recognize that their coupled bi-layer photonic crystal system is equivalent to our coupled resonator system.

(2) $$V=\underset{j}{\prod }2v_{j}$$

The global topological invariant can take on one of three values. When an exceptional point is present, *V* = 0. If only exceptional rings are present, *V* = 1. If any exceptional concentric rings are present, *V* = −1. In our system, we expect an exceptional concentric ring when *κ*_*θ*_, *κ*_h_, *κ*_v_ > 0. Therefore, the sheet of two-fold degeneracy in Figure 1(c) corresponds to an exceptional concentric ring requiring $Z_{3}$ topology with *V* = −1. As shown in Figure 1(d), the exceptional concentric ring can be observed by varying either *κ*_*θ*_ or *κ*_h_ independently, keeping the other parameter constant. This allows us to probe the exceptional concentric ring simply by mapping the *k*-space response of one metasurface (e.g., *κ*_*θ*_ variable, *κ*_h_ constant).

**Figure 2:**
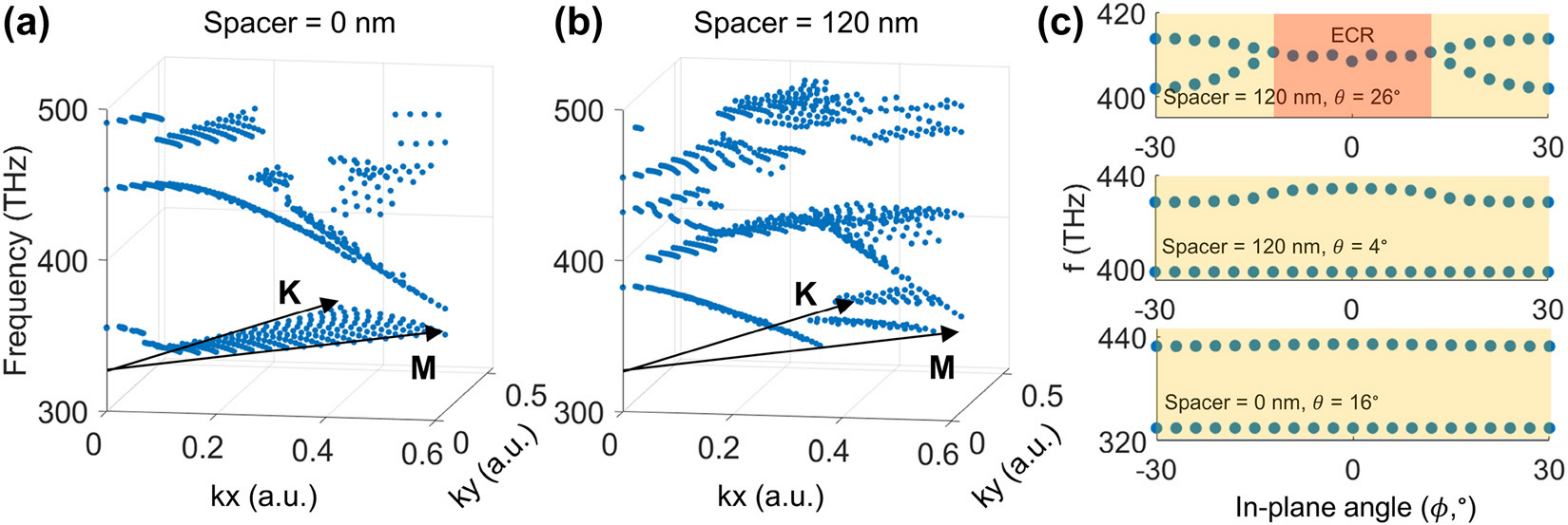
Full-wave simulations of non-Hermitian metasurfaces reveal non-trivial topology. Absorption peaks extracted from full-wave simulations while varying the out-of-plane angle (*θ*) from 0° to 30° and in-plane angle (*ϕ*) from 30° (M point) to 60° (K point) for (a) spacer = 0 nm and (b) spacer = 120 nm. For spacer = 120 nm, an exceptional concentric ring feature is observed. (c) Simulated mode frequencies of the 4 resonator system as the in-plane angle is tuned. For spacer = 120 nm and out-of plane incident angle of 26°, the system undergoes a transition from PT-symmetric (yellow) to exceptional concentric ring (ECR, red) phases.

To verify the analytical model, we performed full-wave simulations to calculate device absorption and experimentally characterized the *k*-space of the fabricated devices. Using full-wave simulations, we characterize the *k*-space response of our metasurface designs. Figure 2(a and b) shows the frequencies of absorption peaks as the out-of-plane angle (*θ*) is swept from 0° (normal incidence) to 30°, and the in-plane angle, defined as *ϕ*, is swept from 30° (M point) to 60° (K point) for silica spacers of 0 and 120 nm. These spacer thicknesses correspond to the PT-symmetric (strong coupling) and PT-broken (weak coupling) regimes, respectively, in the normal incidence case. For spacer = 0 nm, eigenvalue sheets do not coalesce, indicating that the system is always in the PT-symmetric regime. In comparison, for spacer = 120 nm, the system is strongly dependent on in-plane (*ϕ*) and out-of-plane (*θ*) angle. As the out-of-plane angle is increased from 0°, two sheets merge together at 410 THz. Further increasing out-of-plane angle lifts the degeneracy of these sheets – this feature is the exceptional concentric ring observed in the analytical model (1(c and d)). This establishes the equivalence between the considered Hamiltonian and our metasurface design.

We also make use of the in-plane angle (*ϕ*) as a tuning parameter, measuring device response at and between high symmetry points in the *k*-space (M for in-plane angle = 30°, K for in-plane angle = 60° points). In general, the M and K points can exhibit different coupling and damping rates. Thus, the in-plane angle can be used to tune the system in and out of the exceptional concentric ring phase.

Figure 2(c) shows the simulated resonance frequencies as the in-plane angle is tuned for three cases. The first case is when the silica spacer is 120 nm, and the out-of-plane incident angle (*θ*) is 26° away from normal. Here, as the in-plane angle is swept from the M-point to the K-point, the system transitions between the PT-symmetric and exceptional concentric ring phases.

By decreasing the coupling between the horizontal and vertical modes (small *κ*_*θ*_) or increasing the coupling between the plasmonic and photonic modes (large *κ*_h_, *κ*_v_), the device can be pushed to regions where the system is always in the PT-symmetric phase as the in-plane angle is swept. These correspond to the second and third cases in Figure 2(c), respectively. In the second case, coupling between horizontal and vertical modes is decreased by reducing the angle of incidence (spacer = 120 nm, *θ* = 4°). In the third case, coupling between plasmonic and photonic modes is increased by reducing the spacer thickness (spacer = 0 nm, *θ* = 16°).

**Figure 3:**
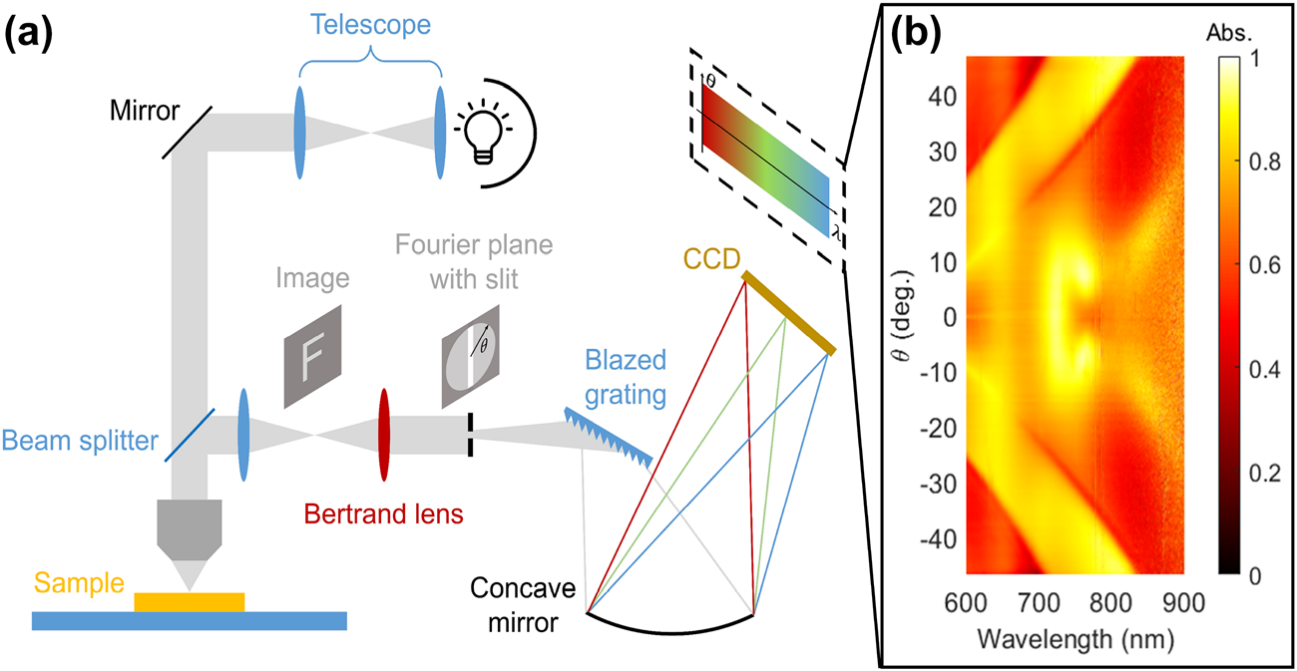
Experiments for Fourier-space imaging for bandstructure measurements. (a) Metasurface sample characterization setup using a Bertrand lens to image the Fourier-plane onto the spectrometer CCD. A tungsten lamp illuminates the sample. (b) Example of a single-shot measurement of the angle-dependent normalized absorption spectrum.

In order to verify our analytical model and full-wave simulations, we fabricated designed metasurfaces with varying silica spacer thickness (see Material and methods). To probe the metasurfaces efficiently, we use a Fourier-space imaging setup that allows for single-shot measurement of angular dependent absorption spectra. Our measurement setup is shown in Figure 3(a). A tungsten lamp is focused onto a metasurface via a 0.72 NA objective lens. Reflected light is collected by a Bertrand lens to image the Fourier-plane onto a CCD spectrometer. In this way, we are able to characterize photonic bandstructure efficiently in single-shot measurements. An example measurement of out-of-plane angle dependent absorption spectra is given in Figure 3(b).

**Figure 4:**
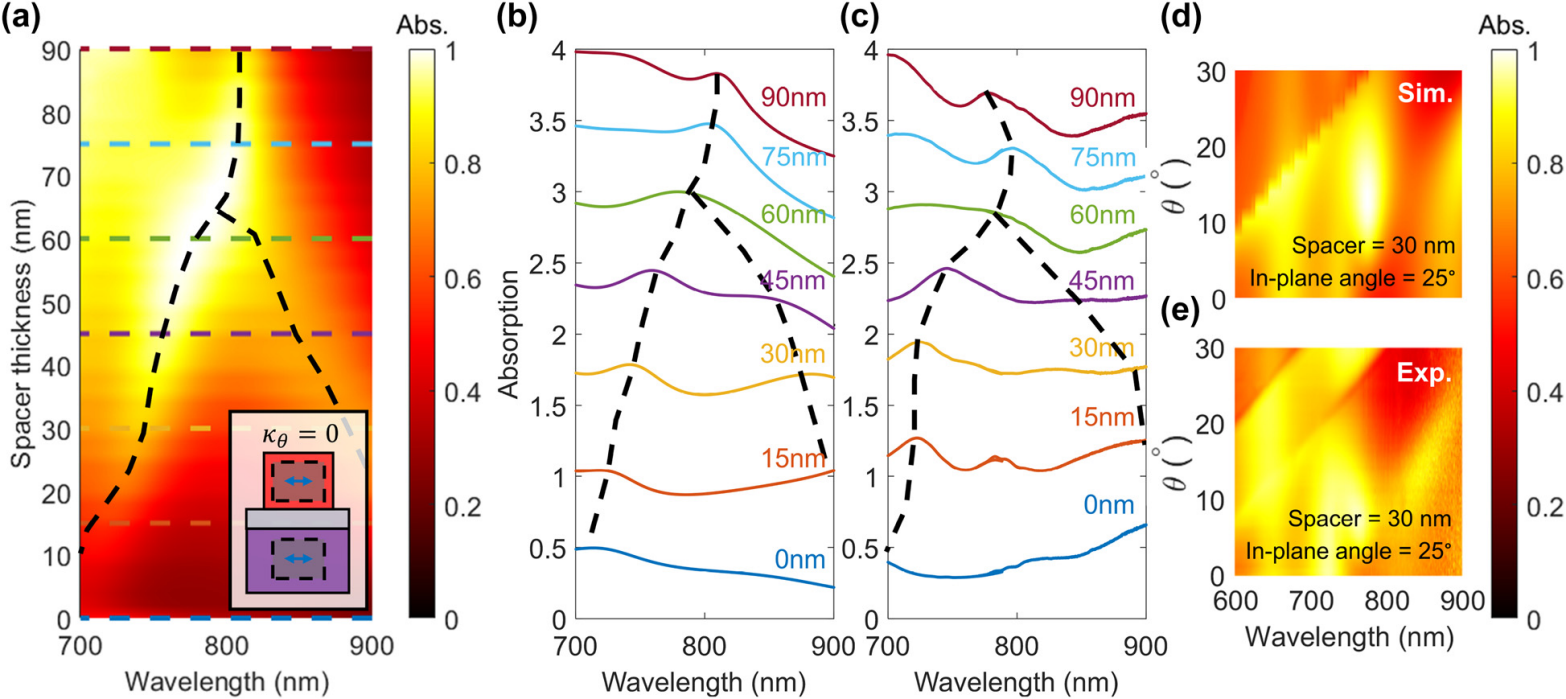
Experimental characterization of non-Hermitian metasurfaces (a) simulated device excited by a normally incident plane wave. Coupling between photonic and plasmonic horizontal modes is tuned by the silica spacer thickness, revealing a PT-phase transition and exceptional point for a 65 nm spacer. Inset: Schematic showing excited horizontal modes and no coupling to vertical modes. (b) Simulation and (c) experimental characterization of metasurfaces excited by normally incident light, confirming the presence of an exceptional point. (d) Simulation and (e) experimental characterization of angle-dependent absorption spectrum show qualitative agreement. Absorption for an in-plane angle of 25° in the PT-symmetric regime (spacer = 30 nm) is plotted.

To confirm the existence of an exceptional point in our non-Hermitian metasurface, we performed measurements on fabricated devices with varying silica spacer thickness (plasmonic-photonic coupling). In this case, we excited the metasurface with normally incident light, so as not to couple to the vertical mode (Figure 4(a) inset). Figure 4(a) shows the existence of a PT-phase transition as the silica spacer is varied from 0 to 90 nm. For low spacer thicknesses, the photonic and plasmonic resonators are strongly coupled (PT-symmetric regime). Thus, the resonance frequencies are split. As the spacer thickness is increased, the resonance frequencies merge at the exceptional point for a 65 nm spacer. Larger spacer thicknesses correspond to the PT-broken (weakly coupled) regime. Figure 4(b and c) give a comparison between simulated and measured absorption spectra from metasurface devices, confirming the existence of the exceptional point. Intermediate peaks in Figure 4(c) occur due to imperfections in measurement of absorption under normal incidence excitation. This results in weak excitation of vertical modes, which is discussed in detail in a previous work [42]. Additionally, a comparison between simulated and measurements of angle-dependent absorption spectrum show qualitative agreement, as shown in 4(d and e). Here, absorption for in-plane angle of 25° in the PT-symmetric regime (spacer = 30 nm) is plotted. The experimental data and simulation results show qualitatively the same behavior for the four modes and their trends. This proves that our simulation model is correctly implemented in experiments. However, the exceptional concentric ring feature is not readily observable in experiments because of the diffraction band and the high absorption losses in the deposited silicon.

## 4 Conclusions

In conclusion, we have demonstrated a topologically non-trivial, non-Hermitian metasurface in the visible. We proposed an analytical four-resonator model for the hybrid photonic–plasmonic resonator system, which displays non-trivial topology. We used a global, non-Hermitian topological invariant capable of detecting exceptional concentric rings and demonstrated $Z_{3}$ topology. We then performed full-wave electromagnetic simulations and experimental characterizations to show that the physical system is consistent with the analytical model. Our results reveal that combining non-Hermiticity with topology relaxes the trade space and simplifies the design of non-Hermitian nanophotonic devices.

## 5 Materials and methods

### 5.1 Simulations

Full-wave electromagnetic simulations are performed using a finite-difference-time-domain solver (Lumerical). Si, SiO_2_, and Al optical constants are obtained from Palik [47] in Figure 2. Silicon optical constants are obtained from ellipsometric measurements in Figure 4(a, b and d). These data are provided in Figure S2 of the Supplementary Material. Simulations performed with normally incident input light use a plane-wave source, and simulations for angular dependent response use the broadband fixed-angle source technique (BFAST).

### 5.2 Device fabrication

The non-Hermitian metasurfaces were fabricated using a Si wafer substrate. Layers of Ti (30 nm), Al (200 nm), SiO_2_ (0–90 nm), and Si (120 nm) were deposited using e-beam evaporation on the Si substrate. The thin layer of Ti serves as an adhesion layer for Al. The Si layer was deposited at a rate of 8 Å/s. Measured optical constants using an ellipsometer (J.A. Woollam M-2000DI) confirm that Si evaporated at such high rates results in a layer with the real part of refractive index similar to that of crystalline silicon (see Figure S2 of the Supplementary Material). The imaginary part of the index exceeds that of crystalline silicon, but these absorption losses do not preclude observation of PT-symmetric behavior. Next, a 13 nm thick Al_2_O_3_ etch mask was patterned using a standard e-beam lithography (Elionix ELS-G100) liftoff process. Silicon nanocylinders were created using a reactive ion etch (Oxford, Plasmalab System 100/ICP 180) with a mixture of C_4_F_8_, SF_6_, CF_4_, and O_2_ at flow rates of 50, 25, 25, and 5 sccm, respectively. The capacitively and inductively coupled RF powers were maintained at 50 and 250 W, respectively.

### 5.3 Optical characterization

Optical characterization is performed in a microscopy setup using a visible spectrometer. Details for Fourier-space spectroscopy are summarized in the text and in Figure 3. For single-shot *k*-space measurements, light reflected from the metasurface sample is collected by a 0.72 NA objective lens. It is then reflected by a beam splitter and passes through an initial lens; the real-space image of the sample is present at the focal plane (focal length (*f*_0_)) of this initial lens (Figure 3). A Bertrand lens (focal length *f*_b_) is placed at a distance *f*_b_ from the focal plane of the initial lens, such that the output of the Bertrand lens is the *k*-space image of the sample. This technique is also known as back focal plane imaging. The *k*-space image passes through an aperture, allowing the incident angles (*θ*) to be resolved. Different in-plane angles (*ϕ*) are probed by rotating the sample.

## Supplementary Material

Supplementary Material Details
