# Supplementary material for: Non-Hermitian metasurface with non-trivial topology
Source: Nanophotonics. 2022 Feb 16;11(6):1159–65. doi: 10.1515/nanoph-2021-0731 (PMC11501583; doi:10.1515/nanoph-2021-0731)
Supplement: Supplementary file 1 — Supplementary Material Details [file j_nanoph-2021-0731_suppl.pdf]

Response Letter

Frank Yang, Ciril S. Prasad, Weijian Li, Rosemary Lach, Henry O. Everitt, and Gururaj V. Naik*

Supplementary Material: Non-Hermitian metasurface with non-trivial topology

https://doi.org/10.1515/sample-YYYY-XXXX
Received Month DD, YYYY; revised Month DD, YYYY; accepted Month DD, YYYY

1 Imaginary part of Hamiltonian eigenvalues

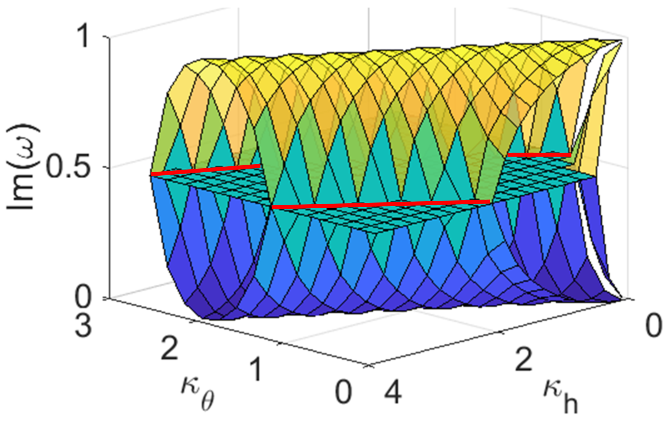

Fig. 1: Hamiltonian eigenvalues. Imaginary part of eigenvalues for representative 4 x 4 Hamiltonian (Equation 1 of main text) of the coupled system, showing exceptional lines (marked in red).

2 Measured optical constants of deposited silicon

***Corresponding author: Gururaj V. Naik,** Department of Electrical & Computer Engineering, Rice University, Houston, TX 77005, USA, e-mail: guru@rice.edu
Frank Yang, Ciril S. Prasad, Weijian Li, Rosemary Lach, Henry O. Everitt, Department of Electrical & Computer Engineering, Rice University, Houston, TX 77005, USA
Ciril S. Prasad, Weijian Li, Applied Physics Graduate Program, Smalley-Curl Institute, Rice University, Houston, TX 77005, USA
Henry O. Everitt, U.S. Army DEVCOM Army Research Laboratory-South, Houston, TX, USA

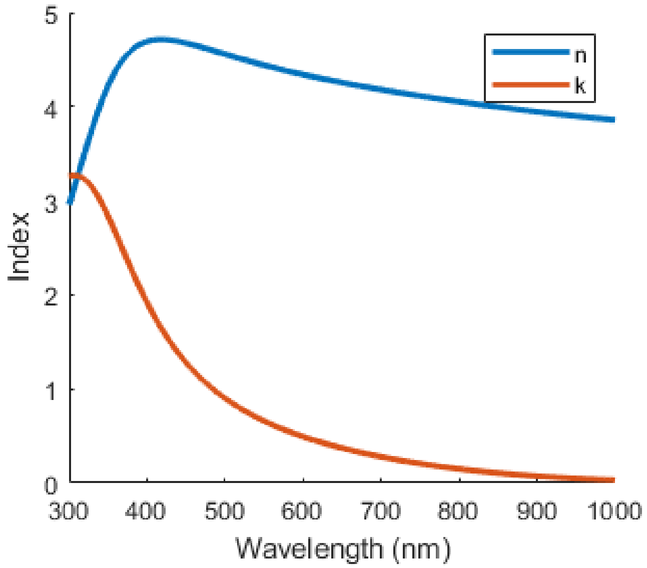

Fig. 2: Measured optical constants of deposited silicon A J.A. Woolam M-2000DI ellipsometer is used to measure optical constants.
